# Advancing Research on Diversity and Resilience in Aging and Dementia: Methodological Challenges and Roadmap Recommendations

**DOI:** 10.1002/alz70860_099973

**Published:** 2025-12-23

**Authors:** Roger A. Dixon, Linzy Bohn

**Affiliations:** ^1^ University of Alberta, Edmonton, AB, Canada

## Abstract

**Background:**

Conceptual developments and research activities pertaining to resilience in aging and dementia are flourishing. However, a variety of methodological challenges have emerged when researchers include dynamic and differential complexities associated with aging and dementia in diverse and intersectional communities. Specifically, resilience patterns are markedly influenced by differing exposures to risk and protection factors as well as disparities in access to social and structural supports for aging brain and cognitive health.

**Objectives:**

(1) Identify key methodological challenges of implementing effective and sensitive research in diverse aging communities. (2) Provide recommendations for precision detection and enhancement of resilience in diverse communities, thereby promoting healthier brain aging and delaying or avoiding dementia.

**Method:**

Consolidating reviews of (1) resilience by the recent NIH resilience collaboratory and other groups; (2) available resilience‐related research with diverse communities and identities; (3) proceedings of the 2024 Montreal collaboratory on developing inclusive approaches to diversity in resilience research.

**Results:**

Four methodological recommendations: (1) Continue active conceptual and operational refinements of resilience, exposure intensity, and disparity measurement; (2) Improve recruitment, retention and reporting practices for participants from diversity communities; (3) Implement designs that are change‐sensitive (longitudinal) and precision‐directed (disaggregation, subtype sensitive) combined with advanced analytics (eg, AI‐related); (4) Explore available aging and dementia databases that include persons from diverse backgrounds with key methodological features (large samples, longitudinal follow‐ups, brain/cognitive data, biomarkers, indicators of risk, exposure and access history).

**Conclusion:**

Roadmap recommendations for integrating diversity in resilience research are graphically illustrated with selected new and published conceptual illustrations and results.

